# Influenza A non-H1N1 associated with acute respiratory failure and acute renal failure in a previously vaccinated cystic fibrosis patient

**DOI:** 10.5935/0103-507X.20180019

**Published:** 2018

**Authors:** Louise Piva Penteado, Cecília Susin Osório, Antônio Balbinotto, Paulo de Tarso Roth Dalcin

**Affiliations:** 1 Academic Course of Medicine, Faculdade de Medicina, Universidade Federal do Rio Grande do Sul - Porto Alegre (RS), Brazil.; 2 Department of Nephrology, Hospital de Clínicas de Porto Alegre, Universidade Federal do Rio Grande do Sul - Porto Alegre (RS), Brazil.; 3 Postgraduate Program in Pneumological Sciences, Faculdade de Medicina, Universidade Federal do Rio Grande do Sul - Porto Alegre (RS), Brazil.; 4 Department of Pulmonology, Hospital de Clínicas de Porto Alegre, Universidade Federal do Rio Grande do Sul - Porto Alegre (RS), Brazil.

**Keywords:** Cystic fibrosis, Influenza A virus, Respiratory insufficiency, Renal insufficiency, Case reports

## Abstract

In the 2014 - 2015 season, most influenza infections were due to A (H3N2) viruses. More than two-thirds of circulating A (H3N2) viruses are antigenically and genetically different (drifted) from the A (H3N2) vaccine component of 2014 - 2015 northern and southern Hemisphere seasonal influenza vaccines. The purpose of this paper is to report a case of seasonal influenza A non-H1N1 infection that occurred in June 2015 in an adult cystic fibrosis patient with severe lung disease previously vaccinated with the anti-flu trivalent vaccine. The patient evolved to respiratory and renal failure (without rhabdomyolysis) and was placed under mechanical ventilation and hemodialysis. The clinical outcome was positive after 39 days of hospital stay. In addition, the patient was clinically stable after 18 months of follow-up. With the recent advances in critical care medicine and in cystic fibrosis treatment, survival with advanced pulmonary disease in cystic fibrosis presents new questions and potential problems, which are still being formulated.

## INTRODUCTION

Influenza, commonly known as the flu, is an acute, contagious respiratory disease caused by influenza A or B viruses. It is estimated that 10% of the world population has at least one annual influenza episode. Patients with chronic disease, especially chronic lung disease, are more susceptible to serious complications caused by influenza.^(1)^

In December 2014, the Center for Disease Control and Prevention (CDC) reported that influenza activity in the northern hemisphere from November 2014 to early 2015 was mainly caused by the influenza A H3N2 virus. In addition, it was evidenced during this period that the effectiveness of the 2014 - 2015 vaccine for the northern hemisphere was very low, which was attributed to the fact that the circulating H3N2 virus was genetically different from the A H3N2 virus component of the vaccine, a fact attributed to a drift of the H3N2 virus after vaccine development.^(2)^ A similar phenomenon occurred with the 2015 vaccine for the southern hemisphere, leading to the low effectiveness of vaccine protection.^(3)^

Recent evidence shows that, in cystic fibrosis (CF), respiratory viruses play an important role in this pathophysiological process, causing pulmonary exacerbations and leading to lung disease progression with increased bacterial adhesion.^(4)^ In particular, influenza virus is associated with more severe exacerbations, leading to prolonged hospitalizations, predisposing to bacterial infections and causing disease progression and marked loss in lung function.^(5)^

This study aimed to report a case of seasonal influenza A non-H1N1 that occurred in June 2015 in a patient with CF who received the trivalent influenza vaccine in April 2015 and developed acute respiratory failure and acute renal failure without rhabdomyolysis after infection.

## CASE REPORT

A male, 39-year-old Caucasian, diagnosed with CF since age 9 years old, homozygous for the F508del mutation, had exocrine pancreatic insufficiency, CF-related *diabetes mellitus*, obstructive azoospermia, chronic lung disease, bronchiectasis and chronic *Pseudomonas aeruginosa* infection. Prior spirometry, with severe airflow limitation and reduction of forced vital capacity, is presented in table 1.

**Table 1:** Spirometry and peripheral oxygen saturation before, immediately after and one year after the event

	August 2014	August 2015	August 2016
VC (L)	2.24	1.52	1.86
VC (predicted %)	54	37	45
FVC (L)	1.98	1.52	1.86
FVC (predicted %)	47	37	44
FEV_1_(L)	1.17	0.75	1.01
FEV_1_(predicted %)	33	21	28
FEV_1_/FVC (%)	61	55	55
SpO_2_ in ambient air	96	87	95

VC - vital capacity; FVC - forced vital capacity; FEV1 - forced expiratory volume in the first second; SpO_2_ - peripheral oxygen saturation. Post-bronchodilator results.

In June 2015, he was admitted to the emergency room of the *Hospital de Clínicas de Porto Alegre*, with continuous fever for 2 days (> 38.5ºC), severe cough, increased volume and purulence of expectoration, prostration, headache and progressive dyspnea. He denied myalgia. He reported that his wife, son and two other family members previously had respiratory symptoms, high fever and myalgias in the last week. He had regularly taken the flu vaccine and had received the trivalent vaccine in April 2015.

At the physical examination, the patient had a blood pressure of 70/45mmHg, a heart rate of 105bpm, a respiratory rate of 30rpm, was cyanotic with accessory breathing muscle and prolonged expiratory time and had a peripheral oxygen saturation (SpO_2_) of 57% in ambient air. Cardiac auscultation revealed a regular two-beat rhythm; lung auscultation indicated vesicular murmur present in both lung fields, with diffuse fine and coarse rales; the patient's abdomen was without changes, and his extremities were well perfused.

**Figure 1:**
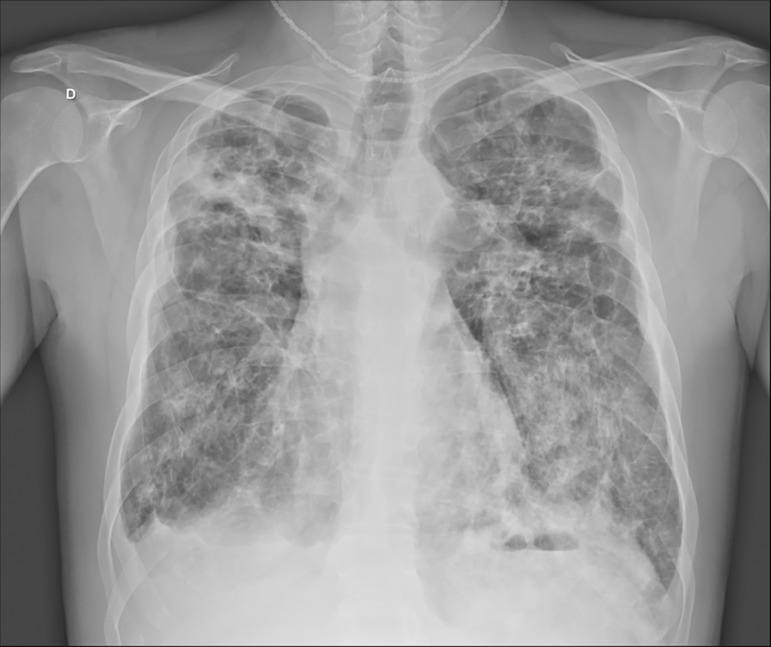
Chest X-ray at hospital admission, showing areas of old fibroatelectasis in the pulmonary apices, related to cystic fibrosis. Extensive alveolar consolidation sites in the lower half of the left lung and bronchiectasis containing large amounts of fluid in the upper right lobe. Obstruction of the lateral costophrenic sinus on the right, due to thickening and/or pleural effusion.

Initial laboratory tests showed a 34% hematocrit, 10.2g/dL hemoglobin, 22,660 leukocytes/mm^3^, 2% band-shaped, 86% segmented, 6% monocytes, 5% monocytes, 1% metamyelocyte, 95mg/dL urea, 2.37mg/dL creatinine (prior it was 0.79mg/dL), 6.6mEq/L potassium, 3.2mmol/L lactate, 145mg/dL C-reactive protein and 189U/L creatine phosphokinase (normal up to 190U/L). Common urine test results revealed citrine yellow urine, 26 leukocytes/µL, 13 red blood cells/µL and negative hemoglobin. An arterial blood gas test revealed 5 L/min oxygen per external nasal catheter, with a pH of 7.21, a partial carbon dioxide pressure (PaCO*_2_*) of 56.1mmHg, 24.4mEq/L bicarbonate, a partial oxygen pressure (PaO*_2_*) of 68.4mmHg and an oxygen saturation of 97%. The patient's chest X-ray is shown in figure 1.

He received volume replacement with saline, correcting the systemic arterial pressure. Antibiotic coverage with piperacillin/tazobactam and intravenous tobramycin was initiated based on the sensitivity test of the last bacteriological examination of the sputum. Still within the first 24 hours of admission, noninvasive ventilation (NIV) and antiviral coverage with oseltamivir were initiated. After 48 hours of hospitalization, he evolved with respiratory failure and was transferred to the intensive care unit (ICU), requiring orotracheal intubation and mechanical ventilation. Initially, the patient was ventilated with controlled pressure, an inspired oxygen fraction (FiO_2_) of 0.40, a positive end-expiratory pressure (PEEP) of 8cmH_2_O, a pressure over PEEP of 14cmH_2_O, a respiratory rate of 18 breaths/minute, a tidal volume of 340mL and a PaO*_2_*/FiO*_2_* of 198mmHg, fulfilling the criteria for acute respiratory distress syndrome.^(6)^

There was also worsening renal function with an increase of creatinine to 5.87mg/dL and oliguria. Dialysis treatment was initiated with continuous hemodialysis. The antibiotic scheme was modified to ciprofloxacin and vancomycin, maintaining piperacillin/tazobactam.

On the fifth day of hospitalization, a viral investigation was performed via polymerase chain reaction from a nasopharyngeal secretion sample, collected at the time of admission. The test was positive for influenza A non-H1N1 (an investigation of a universal gene of influenza A strains was positive, but the swine lines by gene amplification of H1 hemagglutinin and virus nucleoprotein were negative). The use of oseltamivir was maintained for 5 days.

There was favorable evolution and, 7 days after hospital admission, the patient could be extubated and was placed on NIV again. He was then on intermittent dialysis and was discharged from the ICU. In the ward, 1 week after discharge from the ICU, he experienced sudden worsening and was returned to the ICU but was managed with NIV, with a 2-day stay. Angiotomography of the thorax excluded the possibility of pulmonary embolism. The antibiotic scheme was modified to meropenem and polymyxin B (a new sputum culture evidenced multi-resistant *P. aeruginosa*). He experienced progressive clinical improvement of his respiratory condition and resolution of acute renal failure. Hospital discharge occurred 39 days after admission.

On discharge, he received standard treatment for CF and continuous home oxygen therapy and was referred to a pulmonary rehabilitation program. Since the event, he has not required a new hospital stay. The patient's last spirometry is presented in table 1. In December 2016 (18 months after the event), the patient was in pulmonary rehabilitation with oxygen therapy for exercise and at night, and he had been referred for lung transplantation (but not yet active on the list due to clinical stability).

## DISCUSSION

With recent advances in CF treatment and intensive medicine, patients with advanced lung disease are living longer.^(7)^ The present case report shows the evolution of an adult patient with previous severe lung disease, but stable, with acute respiratory failure, sepsis and acute renal failure, triggered by influenza, with a good clinical outcome after intensive support and documentation of clinical stability after 18 months of evolution.

Epidemiological data on influenza 2014 - 2015 show that the vaccine was effective in preventing 19% of influenza visits in all age groups; specifically, 18% for influenza A H3N2 and 45% for influenza B. Despite the low effectiveness of the influenza vaccine, influenza vaccination is recommended for all individuals aged ≥ 6 months.^(2)^

Antiviral medications, especially oseltamivir, are used as adjunctive measures to vaccination in the control of influenza.^(1)^ The physician should consider that influenza activity is widespread; this diagnosis should always be considered in all cases of severe acute respiratory syndrome.^(8-10)^ In the present case, the identification of the influenza A virus was made via molecular biology testing of the nasopharynx aspirate, emphasizing that our laboratory identified the presence of influenza A, excluding the presence of influenza virus H1N1. However, subtyping of the H3N2 virus was not performed. According to epidemiological data from the Department of Health of Rio Grande do Sul^(11)^ and the Ministry of Health of Brazil,^(12)^ the virus with the largest circulation in 2015 was influenza A H2N3. Thus, the viral identification of the reported case most likely corresponds to influenza A H3N2, which explains the disease despite adequate patient vaccination coverage.

Evidence for treating severe influenza is limited; therefore, recommendations on route of administration, dosage and time of use follow the general guidelines. The administration of oseltamivir via the nasogastric or nasoenteric route seems to guarantee adequate systemic absorption in the majority of cases of severe influenza. The standard oseltamivir regimen at a dose of 75mg every 12 hours is recommended. However, although there is no definitive evidence, there are recommendations, in which, depending on clinical judgment, treatment time may be prolonged in severe cases and doses may also be doubled (150mg every 12 hours). In the present case, the treatment was initiated early orally at admission and was subsequently maintained by nasoenteric route, but at usual doses and time.^(8)^

Renal involvement in individuals infected with influenza A is uncommon. Acute renal failure has been most commonly reported in critically ill patients with H1N1.^(13)^ Reports of renal failure in cases of influenza A non-H1N1 are rare. The pathogenic mechanisms for the development of renal injury in influenza A virus infection are not fully understood. Potential causes are rhabdomyolysis, direct renal damage by virus, sepsis-related renal hypoperfusion and disseminated intravascular coagulation syndrome. Among them, rhabdomyolysis has been the most frequently reported cause.^(14)^ In the reported case, there was no evidence of rhabdomyolysis (absence of myalgias, urine was not dark, there was no hematuria on the reactant tape and creatine phosphokinase was normal). The most likely mechanism for renal injury was sepsis and renal hypoperfusion.

## CONCLUSION

After 18 months of follow-up, the evolution of the presented case of cystic fibrosis with advanced lung disease, complicated by infection by influenza A non-H1N1 with respiratory sepsis, acute respiratory failure and acute renal failure was favorable.
